# *Bifidobacterium breve* Attenuates Murine Dextran Sodium Sulfate-Induced Colitis and Increases Regulatory T Cell Responses

**DOI:** 10.1371/journal.pone.0095441

**Published:** 2014-05-02

**Authors:** Bin Zheng, Jeroen van Bergenhenegouwen, Saskia Overbeek, Hendrik J. G. van de Kant, Johan Garssen, Gert Folkerts, Paul Vos, Mary E. Morgan, Aletta D. Kraneveld

**Affiliations:** 1 Division of Pharmacology, Utrecht Institute for Pharmaceutical Science, Faculty of Science, Utrecht University, Utrecht, The Netherlands; 2 Nutricia Research, Utrecht, The Netherlands; National Institutes of Health, United States of America

## Abstract

While some probiotics have shown beneficial effects on preventing or treating colitis development, others have shown no effects. In this study, we have assessed the immunomodulating effects of two probiotic strains, *Lactobacillus rhamnosus (L. rhamnosus)* and *Bifidobacterium breve (B. breve)* on T cell polarization *in vitro*, using human peripheral blood mononuclear cells (PBMC), and *in vivo*, using murine dextran sodium sulfate (DSS) colitis model. With respect to the latter, the mRNA expression of T cell subset-associated transcription factors and cytokines in the colon was measured and the T helper type (Th) 17 and regulatory T cell (Treg) subsets were determined in the Peyer's patches. Both *L. rhamnosus* and *B. breve* incubations *in vitro* reduced Th17 and increased Th2 cell subsets in human PBMCs. In addition, *B. breve* incubation was also able to reduce Th1 and increase Treg cell subsets in contrast to *L. rhamnosus*. *In vivo* intervention with *B. breve*, but not *L. rhamnosus*, significantly attenuated the severity of DSS-induced colitis. In DSS-treated C57BL/6 mice, intervention with *B. breve* increased the expression of mRNA encoding for Th2- and Treg-associated cytokines in the distal colon. In addition, intervention with *B. breve* led to increases of Treg and decreases of Th17 cell subsets in Peyer's patches of DSS-treated mice. *B. breve* modulates T cell polarization towards Th2 and Treg cell-associated responses *in vitro* and *in vivo*. *In vivo B. breve* intervention ameliorates DSS-induced colitis symptoms and this protective effect may mediated by its effects on the T-cell composition.

## Introduction

Inflammatory bowel disease (IBD) is a chronic inflammatory disease that affects the gastrointestinal tract and consists of two major forms, Crohn's disease (CD) and ulcerative colitis (UC). Although the exact mechanisms of IBD development still remain to be elucidated, a feature that is common to IBD pathogenesis is a dysregulated effector T cell response to the commensal microflora [1], [2]. T cells are important components of the adaptive immune system. Upon activation, T cells expand and differentiate into various effector CD4+ T cells such as Th1, Th2, Th17 cells, and Treg cells. The differentiation of these T cell subsets is induced by the specific transcription factors T-bet [3], GATA3 [4], RORγt [5] and Foxp3 [6], [7], respectively.

Until recently, the classical T cell subsets (Th1 and Th2) have been considered the major players during the development of IBD. However, there is an increasing body of evidence showing the importance of the Th17 pathway in IBD [2]. Th17 cells are characterized by RORγt expression and IL17 production [5], [8], and increased Th17 cells have been found in IBD patients [9], [10]. Although the development of Th17 cells is independent of the Th1 and Th2 program, it shares the same requirement for TGFβ with Treg cells [11]. Treg cells have a unique regulatory function by suppressing the activity of other T cell subsets (Th1, Th2 and Th17 cells) and, thereby, helping control autoimmunity [12]. In contrast to Th17 cells, decreased amounts of Treg cells have been found in the peripheral blood of IBD patients as compared to normal controls [13], [14]. In addition, increased apoptosis of Treg cells was found in the inflamed mucosa of IBD patients compared to non-inflamed control colons [15]. Murine models of IBD have further illustrated the protective effects of Treg cells during colitis. Immunodeficient mice that are adoptively transferred with Treg-depleted naïve CD4+ T cells develop spontaneous colitis; in contrast, mice transferred naïve CD4+ T cells combined with Treg cells do not develop colitis [16], [17]. Additionally, Mice lacking interleukin (IL)-10, an important anti-inflammatory cytokine needed for both the induction of Treg cells and their effector function, spontaneously develop colitis [18].

In the last decade, products supplemented with live bacteria, called probiotics, have become increasingly popular [19]. The use of probiotics has been proposed to be beneficial for human health and there is increased interest for their use in IBD. This is due to the beneficial effect of probiotic treatment in other intestinal diseases such as traveler's diarrhea and antibiotic-associated diarrhea [20]. However, the working mechanisms of probiotics still need to be elucidated. Gut-derived bacteria from the genera *Lactobacillus* and *Bifidobaterium* are the most studied probiotics. Diverse effects of the probiotics have been demonstrated using human cell culture systems and animal models and one of the most important effects is their ability to modulate immune responses [21]. Studies using human peripheral blood mononuclear cells (PBMC) have demonstrated the abilities of gut-derived bacteria to modulate T cell polarization by inducing different T-cell subsets including Treg cells in a strain dependent manner [22], [23]. Moreover, two independent clinical studies using two different *Bifidobacteria* strains have demonstrated their immune modulating capacities by both enhancing the TGFβ signaling and increasing peripheral Treg cells numbers [24], [25].

Recently, Plantinga *et al* assessed the cytokine production of PBMC stimulated with two probiotic strains, *L. rhamnosus* and *B. breve*. Exposure to either bacterial strain led to increased IL-10 levels. In addition, exposure to *B. breve* led to a reduction of IFNγ production, a Th1-associated cytokine, as compared to the *L. rhamnosus* [26]. In this study, we further investigated the same probiotic strains by examining their effects on CD4+ T cell differentiation both *in vitro* and *in vivo*. We demonstrated that both strains had the ability to shift CD4+ T cell polarization in stimulated PBMCs away from Th17 cell development towards Th2 differentiation. In addition, *B. breve* induced the development of Treg cells while decreasing the development of Th1 cells. Administering these bacterial strains in the DSS-induced colitis model showed that while *L. rhamnosus* had little effect on disease severity, *B. breve* ameliorated DSS-induced colitis, increased Treg- and Th2-associated responses and locally reduced CD4+RORγt+Foxp3- T cells while simultaneously increasing CD4+ RORγt-Foxp3+ T cells.

## Methods and Materials

### Human peripheral blood mononuclear cell stimulations

Human PBMCs were isolated from buffy coats, which were obtained from the Sanquin blood bank (Utrecht, the Netherlands). The cell fraction containing PBMCs was obtained by density centrifugation of 1∶3 diluted buffy coats on Ficoll-Paque PLUS (GE Healthcare, Eindhoven, the Netherlands). Subsequently, the obtained cells were washed with phosphate buffered saline (PBS; Lonza Verviers SPRL, Verviers, Belgium) and the erythrocytes were lysed using sterile lysis buffer (0.15M NH_4_Cl, 0.01 M KHCO_3_ and 0.1mM EDTA, pH 7.4). After lysis, the remaining cells (PBMCs) were washed again with PBS supplemented with 2% heat-inactivated Fetal Calf Serum (FCS; Lonza Verviers SPRL, Verviers, Belgium) and resuspended in Roswell Park Memorial Institute (RPMI) 1640 medium (Lonza Verviers SPRL, Verviers, Belgium) supplemented with 2.5% FCS, 1% penicillin/streptomycin, 1 mM pyruvate and 50 µg/ml gentamicin.

A total of 10^5^ PBMCs were incubated either with anti-CD3 (Sanquin, Amsterdam, the Netherlands) alone (at a final concentration of 1∶10.000) or in combination with *L. rhamnosus* or *B. breve.* Both *L. rhamnosus* (NutRes 1 formerly known as NumRes 1) and *B. breve* (NutRes 204 formerly known as NumRes 204) were provided by Danone Research BV (Wageningen, the Netherlands) as live bacteria in a 20% glycerol stock. The bacteria:PBMC ratio was 20∶1 and incubated in 96-well plates (Greiner bio-one, Stonehouse, UK) at 37°C for 48 hours or 7 days.

### Experimental colitis and administration of probiotics

Female C57BL/6 mice were purchased from Charles River Laboratories (Maastricht, the Netherlands). All mice were used at 8–12 weeks of age and were housed under standard conditions in the animal facilities at Utrecht University.

Experimental colitis was induced by adding 1.5% DSS to the drinking water for 5 days. 10^9^/dose of *L. rhamnosus* or *B. breve* probiotics were administrated by oral gavage every two days, starting 9 days prior to the DSS treatment and continued to the end of the experiment. Colitis development was monitored by measuring the weight and the fecal condition. The fecal condition was measured on day 0, 3 and 5. On day 6, the mice were sacrificed and the colons and Peyer's patches were isolated for further analysis.

The severity of the colitis was determined by calculating the body weight change, feces condition and the colon length. The body weight change was determined by calculating the percentage of weight change relative to the starting weight before DSS treatment on day 0. The fecal condition score was determined using two parameters: stool consistency (0  =  normal, 1  =  soft with normal form, 2  =  loss of form/diarrhea) and fecal bleeding (0  =  no blood, 1  =  blood observation using Colo-rectal Test kit (Axon Lab AG, Germany), 2  =  blood observation without test).

After sacrificing the mice, the colons were excised between the ileocaecal junction and rectum and were prepared for histological evaluation. The colon was opened longitudinally, placed on a piece of blotting paper, and fixed in 10% formalin. After fixing, the colons were rolled, paraffin-embedded, and sectioned (5 µm). Two researchers assessed general inflammatory features blindly after staining sections with hematoxylin and eosin according the assessment system described below. Assessments included four pathological criteria: the extent of cellular infiltration (0: no infiltration, 1: infiltration between the crypts, 2: infiltration in the submucosa, 3: infiltration in the muscularis externa, 4: infiltration in entire tissue); cover area of cellular infiltration in the region (0: no infiltration, 1: < 25%, 2: 25%–50%, 3: 50%–75%, 4: >75%); loss of crypts (0: no damage, 1: 30% shortening of crypts, 2: 65% shorting of crypts, 3: total loss of crypts, 4: loss of entire epithelial layer); extent of crypts loss in the region (0: no crypt loss, 1: < 25%, 2: 25% – 50%, 3: 50%–75%, 4: > 75%).

### Ethics statement

All experiments were performed in accordance with the guidelines issued by the Dutch ethics committee for animal studies. The protocol was specifically approved by the ethics committee for animal studies of Utrecht University (DEC approval number 2009.II.06.046). All efforts were made to minimize suffering.

### Immunohistochemical staining

A subset of the mice from each group was examined using immunohistochemistry. After sacrificing the mice, the colons were opened longitudinally and half of each colon was fixed in 10% formalin, rolled, paraffin-embedded, and sectioned (5 µm). The sections were subjected to a heat-induced epitope retrieval step. Slides were washed with PBS and blocked with rabbit or goat serum before an overnight incubation (4°C) with primary antibodies against Ly-6B (AbD Serotec, Dusseldorf, Germany), RORγt (eBioscience San Diego, CA USA) or Foxp3 (eBioscience San Diego, CA USA). For detection, biotinylated goat anti-rat (Dako, Glostrup, DK) secondary antibodies were administered followed by incubation with peroxidase-labeled streptavidin (Vectastain EliteABC kit, Vector, Burlingame, CA USA). The peroxidase activity was visualized using the substrate, DAB (Sigma, Gillingham, UK). The cell nuclei were visualized by a short incubation with Mayer's hematoxylin (Klinipath, Duiven, the Netherlands). Background staining was determined by substituting the primary antibody with a rat IgG isotype control (Abcam, Cambrige, UK).

The number of Foxp3+ cells was quantified by counting positive cells within the lamina propria area excluding the induced and tertiary lymphoid follicle regions. The density of RORγt+ cell was determined as follows: RORγt+ cells were counted in colonic patches and quantified as a function of 0.01mm^2^ colonic patch area.

### MPO measurement

A subset of the mice from each group was used to determine the MPO concentration in the colon. After sacrificing the mice, the colons were opened longitudinally and half of each colon was transferred into RIPA buffer (Thermo Scientific, Rockford, IL USA) and homogenized using a Precellys 24-Dual homogenizer (Precellys, Villeurbanne, France). The homogenates were centrifuged at 14000 rpm for 10 minutes at 4°C and the MPO concentration in the supernatant was measured using an ELISA kit according to the manufacturer's protocol (Hycult biotech, Uden, the Netherlands).

### Real-time PCR

A subset of the mice from each group was used to determine the mRNA expression of a selection of genes in the colon. After sacrificing the mice, Total RNA of 1 cm distal colon pieces was isolated using the RNAeasy kit (Qiagen, Germantown, MD USA) and, subsequently, reverse transcribed into cDNA using the iScript cDNA synthesis kit (BioRad, Hercules, CA USA). Real-time PCR quantification was performed using the iQ SYBR Green super mix kit (BioRad, Hercules, CA USA) with the CFX 96 Real-time system (BioRad, Hercules, CA USA) and the RNA expression was determined using built-in detection system of CFX 96 Real-time system (BioRad, Hercules, CA USA). The RNA expression value and normalized gene expression (ΔΔC_T_) value was calculated using the built-in gene expression analysis module in CFX Manager software version 1.6. The sequence of specific primers for T cell transcription factor genes and the gene for the household protein *ribosomal protein S13* (*Rps13*) are listed in Table 1. The primers for the cytokines: *interferon gamma (Ifnγ), Il12p35, Il4, Il5, Il13, Il23p19, Il17, Il6, Tgfβ* and *Il10* were purchased from SABioscience (Frederick, MD USA). The final data for the target samples were normalized against the internal control *Rps13*.

**Table 1:** qPCR primer sequences.

Primer Sequence 5'–>3'		
	Forward primer	Reverse primer
*Gata3*	GCGGTACCTGTCTTTTTCGT	CACACAGGGGCTAACAGTCA
*Foxp3*	CACTGGGCTTCTGGGTATGT	AGACAGGCCAGGGGATAGTT
*Rorc*	TGCAAGACTCATCGACAAGG	AGGGGATTCAACATCAGTGC
*Rps13*	GTCCGAAAGCACCTTGAGAG	AGCAGAGGCTGTGGATGACT

### Intracellular staining for cytokines and transcription factors

The isolated human PBMCs were incubated for 48 hour or 7 days as described. The PBMCs, which were incubated for 48 hours, were stained first extracellularly with antibodies for CD4 and CD69, followed by intracellular staining for GATA3, RORγt, FOXP3 and T-bet. The Fluorescent Minus One (FMO) control of each marker was determined by taking out the indicated marker antibody during the staining of control human PBMCs. In addition, the possible background of each marker antibody within CD4 cells was determined by substituting the indicated antibody with an appropriate isotype antibody with a matching fluorescent label.

The PBMCs, which were incubated for 7 days, were provided with fresh culture medium for 24 hours and then subsequently stimulated with PMA (50ng/ml) and ionomycin (750ng/ml) in the presence of Brefeldin A (eBioscience, San Diego, CA USA) for 4 hours. After stimulation, PBMCs were first stained extracellularly with anti-CD4, followed by intracellular staining for IL-4, IL-17, IL-10 and IFNγ. The FMO controls and isotype controls of these marker antibodies were also assessed as described in the previous paragraph.

Peyer's patches isolated from the mice of experimental colitis study were prepared as single-cell suspensions by passing through a 0.75 µm cell strainer. Cells were first stained extracellularly with antibodies for CD4, followed by intracellular staining for Foxp3 and RORγt. The FMO controls and isotype controls of these marker antibodies were examined in mLN cells obtained from non-treated mice. All antibodies and intracellular staining buffers were obtained from eBioscience (San Diego, CA USA). All samples were read on a BD FACSCanto II (BD Biosciencse, Franklin Lakes, NJ USA) and the data were analyzed using BD FACSDiva software (BD Biosciences). The (activated) T cells were determined by gating on CD4+ (CD69+) cells. Subsequently, the different T cells subsets were defined on the found T cell subset associated transcription factors and the specific cytokine producing T cells were found by gating on appropriated cytokines.

### Statistical analysis

Means with SEM are represented in each graph. Statistical analysis was performed using GraphPad Prism version 5.0 for windows (GraphPad Software, San Diego, CA USA). P-values were calculated using either the 2-way ANOVA followed by Bonferroni post-tests or a Mann-Whitney test. P-values considered as significant are indicated as ***<0.001, **<0.01, and *<0.05.

## Results

### *L. rhamnosus* and *B. breve* reduce Th17 differentiation in PBMCs

**Figure 1:**
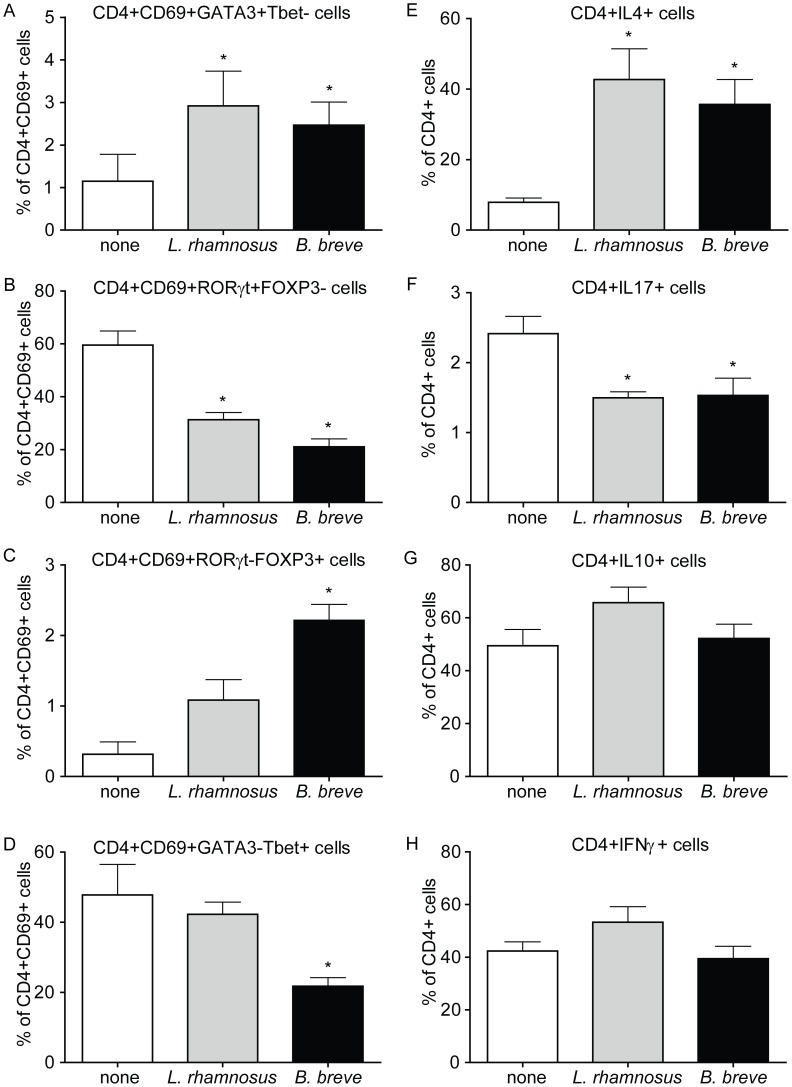
*L. rhamnosus* and *B. breve* alter T cell differentiation in human PBMCs. PBMCs were stimulated with anti-CD3 alone (white bars), with a combination of anti-CD3 and *L. rhamnosus* (grey bar) or a combination of anti-CD3 and *B. breve* (black bar) for 48 hours or 7 days. A–D) The percentages of Th2 (GATA3+Tbet-), Th17 (RORγ+FOXP3-), Treg (RORγ-FOXP3+) or Th1 (GATA-Tbet+) cells within the activated T cells (CD4+CD69+) in the PBMCs were determined after 48 hours of incubation. Percentages within activated CD4+CD69+ T cell population are shown. E–H) The percentages of cytokines (IL10, IL17, IL4 or IFNγ) producing CD4+ T cells in the PBMCs were determined after 7 days of incubation. Percentages within CD4+ T cell population are shown. The Results are expressed as mean + SEM, n = 3, * p<0.05.

To assess the immunomodulatory capacity of the bacterial strains, PBMCs were stimulated with a combination of anti-CD3 together with *L. rhamnosus* or *B. breve*, and the different T cells subsets were analyzed using flow cytometry. Differences were found in the T cell subtype composition within the activated CD4+CD69+ T cells (Figure S1 and Figure 1A – D). Both strains significantly increased Th2 (CD4+CD69+GATA3+ Tbet-; Figure 1A) and decreased Th17 (CD4+CD69+RORγ+FOXP3-; Figure 1B) cell subsets. Incubation with *B. breve*, but not *L. rhamnosus*, led to a significantly increased Treg (CD4+CD69+RORγ-FOXP3+; Figure 1C) and decreased Th1 cell (CD4+CD69+ GATA3+Tbet-; Figure 1D) subsets. To further confirm the changes in T cell subsets, the IL-4, IL-17, IL-10 and IFNγ producing CD4+ T cells within total PBMCs were analyzed after 7 days of stimulation (Figure S2 and Figure 1E – H). Cytokine expression of CD4+ T cells stimulated with anti-CD3 and the bacteria mirrored the results seen with the transcription factors. Both *L. rhamnosus* and *B. breve* significantly increased the population of CD4+IL-4+ T cells and decreased the population of CD4+IL17+ T cells (Figure 1E and F). No changes were observed in the CD4+IL-10+ and CD4+IFNγ+ T cell populations for both bacteria (Figure 1G and H). The results of FMO controls and isotype controls indicate that the staining antibodies were working sufficiently and that we used proper gate-settings (Figure S1 and S2).

These data indicate that *L. rhamnosus* as well as *B. breve* are able to limit the differentiation of CD4+ T cells *in vitro* towards Th17 cells. Additionally, *B. breve* induced *de novo* Treg induction and reduced Th1 cells.

### Intervention with *B. breve*, but not *L. rhamnosus*, ameliorates DSS-induced colitis

**Figure 2:**
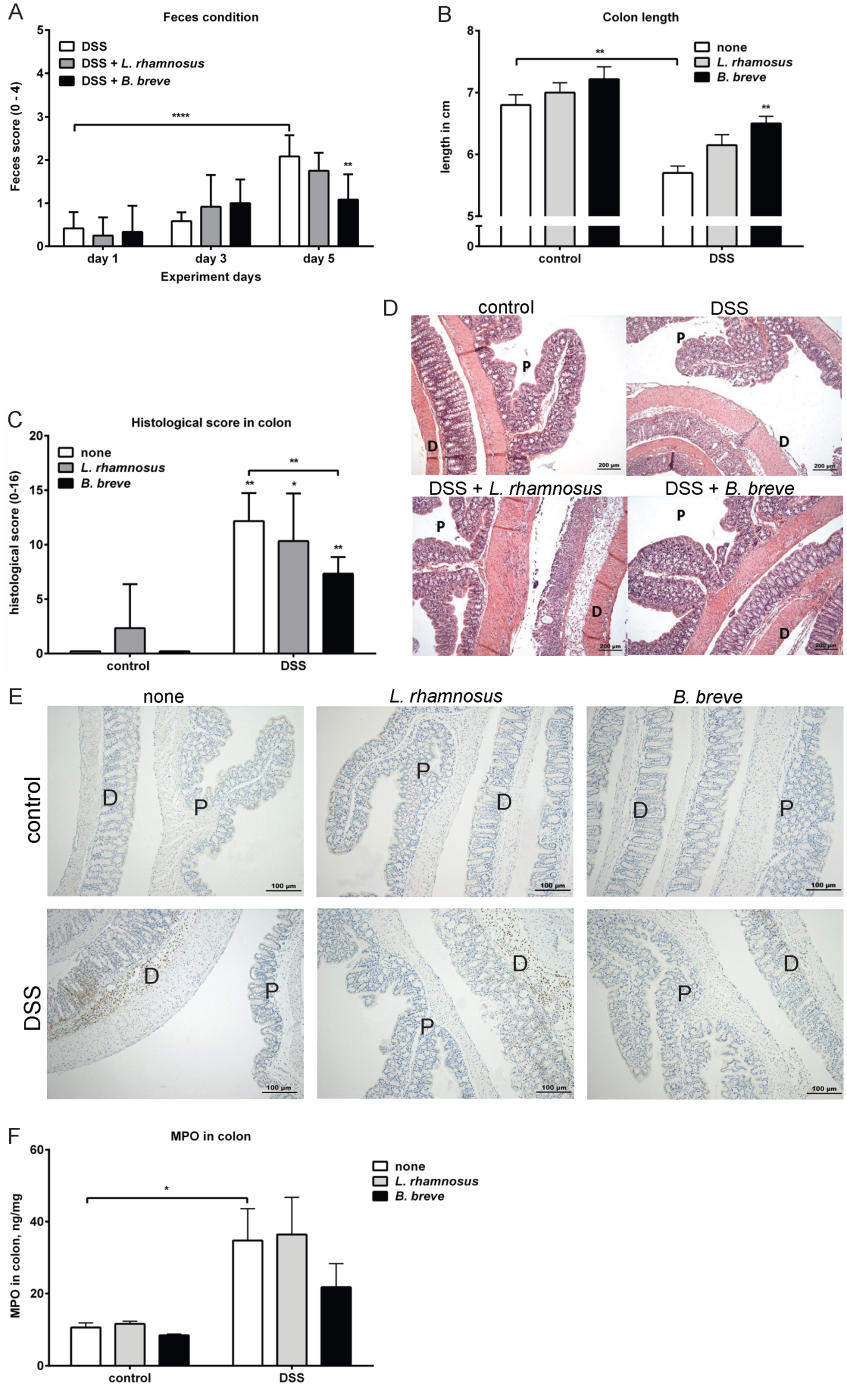
*B. breve*, but not *L. rhamnosus*, ameliorates DSS-induced colitis. C57BL/6 mice with or without probiotics treatment received either normal drinking water or drinking water with DSS for 5 days. A) The fecal condition was calculated on day 0, day 3 and day 5 after DSS treatment. On day 6, the mice were sacrificed and B) the colon length of each mouse was measured. Results are expressed as mean ± SEM, n = 6 mice per group, pooled from two independent experiments. Colons were collected and examined for histological score as described in materials and methods. C) The histological scoring graph and D) representative H&E staining photos are shown. Results are expressed as mean + SEM, n = 3 mice per group, pooled from two independent experiments. E) The presence of Ly-6B+ cells was visualized in the proximal (p) and distal (d) colons using immunohistochemistry. The pictures are representative of 3 separate mice per group obtained from two experiments. F) The concentration of MPO was measured in colon homogenates of each group. Results are expressed as mean + SEM, n = 4 mice per group, pooled from two independent experiments. * p<0.05; ** p<0.01.

To study the effect of the *L. rhamnosus* and *B. breve* strains *in vivo*, the murine DSS-induced colitis model was used. Mice received *L. rhamnosus* or *B. breve* 9 days prior to colitis induction and the bacterial administration was continued until the end of the experiment. Control mice receiving bacteria did not display any clinical changes (data not shown). DSS treatment increased feces condition score, histology score and mildly reduced body weight and the colon length. Intervention with *B. breve*, but not *L. rhamnosus* led to improvement of feces condition and to a significant reduction of DSS-induced colon shortening, colon epithelial damage and cellular infiltration as compared to mice with DSS treatment alone (Figure 2A–D).

In order to visualize changes in infiltrating inflammatory cells in the colon after DSS treatment, immunohistochemistry was employed to determine the number of cells expressing Ly-6B, which is expressed on the surface of neutrophils and inflammatory macrophages [27]. DSS treatment significantly enhanced the infiltration of Ly-6B+ cells. Mice treated with DSS and *B. breve* intervention tended to have reduced amounts of Ly-6B+ cells in the colon (Figure 2E). Consistent with the Ly-6B staining, quantification of MPO concentration (an indicator for neutrophil influx) in the colon showed that DSS treatment significantly increased the MPO concentration in colon of colitis mice. Intervention with *B. breve* reduced the MPO expression by approximately 35%, although no significant different was determined (Figure 2F).

These data indicate that *B. breve* intervention leads to improvements in the outcome of DSS-induced colitis in mice.

### *B. breve* intervention enhances the mRNA expression of Th2- and Treg-associated cytokines in distal colon

As both *L. rhamnosus* and *B. breve* were able to alter T cell differentiation *in vitro*, we investigated if *L. rhamnosus* and *B. breve* induced similar changes *in vivo* during colitis. DSS-induced colitis, on its own, significantly increased the mRNA expression of *Ifnγ, Il6, Il17* and *Tgfβ* as compared to controls. *L. rhamnosus* intervention did not modulate the transcription of cytokines in healthy control mice nor DSS-treated mice, except for a significant increase of *Il5* in DSS-treated mice (data not shown).

**Figure 3:**
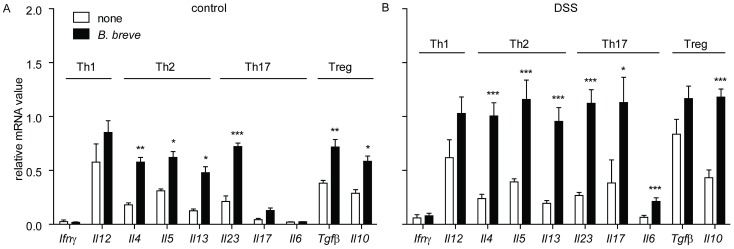
*B. breve* intervention changes mRNA expression of Th2-, Th17- and Treg- associated cytokines in the colon. The mRNA expression of Th1- (*Ifnγ* and *Il12*), Th2- (*Il4, Il5* and *I13*), Th17- (*Il23* and *Il17*) and Treg- (*Tgfβ* and *Il10*) associated cytokines was quantified in the distal colons of both A) healthy and B) DSS-treated mice with or without *B. breve* intervention. Results are expressed as mean + SEM, n = 5 mice per group, pooled from two independent experiments. * p<0.05; ** p<0.01; *** p<0.001.

*B. breve* administration in healthy control mice, on the other hand, significantly increased mRNA transcription of Th2- (*Il4*, *Il5* and *Il13*) and Treg- (*Il10* and *Tgfβ*) associated cytokines as well as *Il23* in the colon. In contrast, Th1- (*Ifnγ* and *Il12*) associated cytokines (Figure 3A) were unaffected. *B. breve* intervention of DSS-treated mice induced a similar mRNA cytokine expression pattern in the colon as healthy control mice with *B. breve intervention*. However, the expression was more pronounced and significantly increased *Il6* and *Il17* mRNA expression levels were observed (Figure 3B).

These results demonstrate that *B. breve* intervention alters mRNA expression patterns in the colon and increased the mRNA expression of *Il6* and *Il17*, and Th2 and Treg-associated cytokines.

### *B. breve* intervention leads to increased numbers of Foxp3+ cells in the colon and altered Treg and Th17 cell populations in the Peyer's patches during colitis

**Figure 4:**
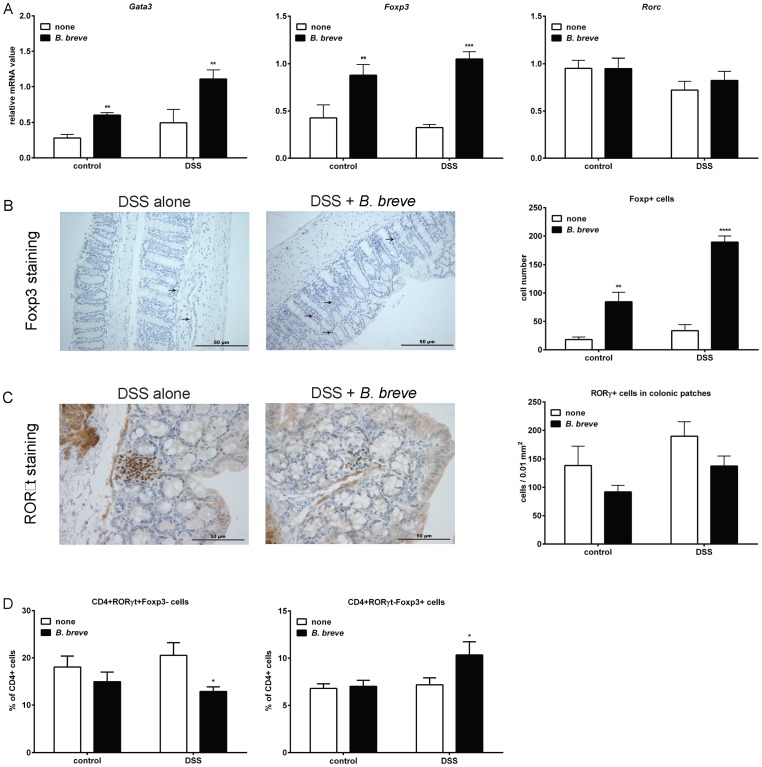
*B. breve* intervention leads to increased numbers of Foxp3+ cells in the colon and Peyer's patches. A) The mRNA expression of Th2- (*Gata3*), Th17- (*Rorc*) and Treg- (*Foxp3*) associated transcription factors was quantified in the distal colons of both healthy and DSS-treated mice with or without *B.breve* intervention. Results are expressed as mean + SEM, n = 5 mice per group, pooled from two independent experiments. B) Foxp3+ cells cells were visualized in the colon of DSS-treated mice with or without *B. breve* intervention using immunohistochemistry. The number of Foxp3+ cells was determined as described in the materials & methods and shown in the graph, The pictures are representative of n = 3 mice per group obtained from two independent experiment. C) RORγt+ cells were visualized in the colon of DSS-treated mice with or without *B. breve* intervention using immunohistochemistry. The number of RORγt+ cells was determined as described in the materials & methods and depicted in the graph. D) The percentage of Th17 cells (CD4+RORγt+Foxp3-) and Treg cells (CD4+RORγt-Foxp3+) was determined in the Peyer's patches obtained from both healthy and DSS-treated mice with or without *B. breve* intervention. Percentages within CD4+ T cell population are shown. Results are expressed as mean + SEM, n = 6 mice per group, pooled from two independent experiments. * p<0.05, ** p<0.01; *** p<0.001.

As intervention with *B. breve* led to significant changes in cytokine transcription that were indicative for skewing in the T cell response towards a Th2 and Treg response combined with a Th17 response, we assessed the mRNA expression of Th17-, Th2- and Treg-associated transcription factors; *Rorc, Gata3* and *Foxp3*, respectively, in the colon. Significantly increased *Gata3* and *Foxp3* mRNA expression levels were detected in both healthy and DSS-treated mice receiving *B. breve*, while no different was detected for *Rorc* expression in both healthy and DSS-treated mice receiving *B. breve* (Figure 4A).

To determine whether increases in the regulatory T cell response caused by *B. breve* intervention were also reflected by an increased number of Foxp3+ cells in the colon, we visualized and quantified colon Foxp3+ cells using immunohistochemistry. Indeed, increased numbers of Foxp3+ cells were found in the colon of DSS-treated mice with *B. breve* intervention (Figure 4B).

It has been shown that the conditions, which favor Treg development, naturally antagonize Th17 polarization [28]. Since Th17 cells express the transcription factor RORγt [5], we also examined the numbers of RORγt+ cells in the colon using immunohistochemistry. RORγt+ cells were found primarily in the lymphoid follicles and we analyzed the number of these cells per 0.01 mm^2^ colonic patch. When analyze the effect of *B. breve* intervention on the number of RORγt+ cells in colonic patches, taking the *B. breve* intervention and exposure to water or DSS together, a trend of decreasing the amount of RORγt+ cells was observed in mice with *B. breve* (Two way ANOVA: F_1,8_ = 4,29 p = 0.07, Figure 4 C).

Although only a trend in reducing the number of RORγt+ cells by *B. breve* was observed in the colonic patches, analysis of CD4+ T cells within GALT, namely the Peyers patches of the small intestine, using flow cytometry revealed that *B. breve* intervention significantly decreased the Th17 (CD4+RORγt+Foxp3-) cell subset in Peyer's patches and significantly increased the Treg (CD4+RORγt-Foxp3+) cell subset (Figure 4D and Figure S3).

These results indicate that *B. breve* intervention is capable of increasing the Treg cell population and decreasing Th17 cells in the GALT during colitis.

## Discussion

While the incidence rate of IBD has increased [29], there is still no curative therapy for IBD, and the treatments that do exist focus mainly on relieving symptoms and often lead to unwanted side-effects [30]. In the last decade, probiotics, defined as “live microorganisms that when administrated in adequate amounts, confer a health benefit on the host”, have been proposed as potential candidates for IBD treatment. The increased interest in the immunomodulatory properties of specific probiotic strains stems from the success of using probiotics to treat a varied number of intestinal diseases [20]. Since a dysregulated T cell response is a common feature in IBD [31], we assessed the capability of two probiotic strains, *L. rhamnosus* and *B. breve* to modulate the development of different T cell subsets *in vitro*, using PBMCs isolated from healthy volunteers. In addition, the effect of these specific bacterial strains on the experimental colitis and the development of different T cell subset *in vivo* have been assessed. We hypothesize that these specific gut-derived bacterial strains could have protective effects on experimental colitis via their capability to modulate the development of different T cell subsets.

Our results were generally consistent with the results from previous study by Plantinga *et al* concerning the same bacteria [26]. In our study, significantly decreased CD4+CD69+RORγt+FOXP3 and CD4+IL-17+ T cell subsets were observed in PBMCs stimulated with both bacteria, however, only *B. breve* stimulation led to a reduction of the Th1 cell subset. In addition, we found *B. breve* stimulation significantly increased the FOXP3+ Treg cell subset, which is often associated with anti-inflammatory effects [12], suggesting an anti-inflammatory property of this bacterial strain.

The increased Th2 cell subset found in PBMCs stimulated with *B. breve* may contribute to the decreased Th1 cell subset due to the mutual antagonizing effects of Th1 and Th2 cells on each other [32]. Increased Th2 cells and CD4+IL4+ T cell subsets were also observed in PBMCs stimulated with *L. rhamnosus*, but no change in the Th1 cell subset was seen suggesting that *L. rhamnosus* may have a different T cell modulating mechanism.

The capability of *B. breve* to alter T cell differentiation by inducing Treg cell and reducing Th17 cell development *in vitro* indicates that using this specific bacterial strain *in vivo* may have a protective function in IBD. Murine colitis models are a useful tool to examine the clinical efficacy and possible working mechanism of probiotics in the development of IBD. A multitude of *Bifidobacteria* strains have shown protective effect in colitis models. For example, a mixture of probiotics including *Bifidobacterium longum* induces Treg cell expansion and prevents trinitrobenzene sulfonic acid (TNBS)-induced colitis in mice [33]. In addition, prior administration of a probiotic mixture, including four *Bifidobacteria* strains, to DSS-treated mice also demonstrated protective effects [34], [35].

Here, we tested the effect of *B. breve* administration in a DSS-induced colitis model. For a long time, the DSS-induced acute colitis model was regarded by some as an erosive, self-limiting model of colonic injury and inflammation. A previous study has demonstrated that T cells are not necessary in DSS-induced colitis [36]. However, recent studies show that bacteria penetrated the mucosa layer before inflammation in acute colitis model [37] and microflora is necessary during the development of DSS colitis [38]. Penetration of bacteria in the mucosal layer will lead to the activation of resident innate immune cells that in turn can lead to an adaptive immune response where T cells are involved. Indeed, we have recently demonstrated that antigen-specific T cells develop during the acute stage of DSS-induced colitis [39]. In addition, it has been shown that transient Treg depletion leads to increased severity of DSS colitis [40]. We hypothesized that the induction of Treg cells in the intestinal mucosa by intervention with *B. breve* could induce protective effect during DSS-induced colitis. The DSS colitis model is, thus, a valuable model to investigate the rise of T cell associated responses during intestinal inflammation mimicking early IBD. Altogether, T cells can affect the development of acute colitis, although the specific mechanism still needs to be investigated.

In this study, our data show that intervention with *B. breve* is beneficial in DSS-induced colitis by improving the weight loss, fecal condition, colon histology score which includes epithelial damage and cellular infiltration and colon shortening. DSS-induced colitis is often associated with increased MPO activity, which is indicative for an increased number of infiltrating neutrophils [41]. In line with this finding, increased numbers of LyB6+ cells and increased MPO levels were found in the colons of DSS-treated mice. DSS-induced enhancement of MPO expression was decreased by 35% due to *B. breve* intervention, although this did not reach significance. Intervention with *L. rhamnosus* did not affect the DSS-induced colitis, which is similar to results found in experiments using *Lactobacillus rhamnosus GG* performed by Mileti *et al* [42]. A possible explanation could be that *L. rhamnosus* is less able to modify the T cell composition as compared to *B. breve*. it has been postulated that it is essential to target both Th1 and Th17 cells for treatment for CD, the major form of IBD [43]. The fact that *L. rhamnosus* is not as protective for DSS-induced colitis as *B. breve* could be explained by data from the *in vitro* experiments that show exposure to *B. breve* reduced both Th1 and Th17 cell subsets, whereas exposure to *L. rhamnosus* only reduced the Th17 cell subset. In addition, *B. breve* increased the expression of Treg cell–associated cytokines and transcription factors *in vivo*, while *L. rhamnosus* did not induce any of these changes.

Analysis of mRNA expression in the colon showed increased expression of Th2 (*Il4, Il5 and Il13*)- and Treg (*Il10*)-associated cytokines in both healthy and DSS-treated mice with B. breve intervention. An increased Th2 response often results in a decreased Th1 response due to the mutual antagonizing effects of Th1 and Th2 cells on each other [32]. Treg cells are able to repress the activity of other T cell subsets to induce an anti-inflammatory effect [12]. There are two major regulatory T cell populations, namely Foxp3+ Treg and IL10-producing type 1 regulatory T (Tr1) cells that are know to maintain intestinal homeostasis [44]. Therefore, it can be concluded that besides Foxp3+ Treg cells, Tr1 cells could also be involved. Interestingly, the increased *Il10* expression in the colon is in line with recent findings, which demonstrated an increased number of IL10 producing Tr1 cells in the colon after *B. breve* intervention [35].

Next to the increased Th2- and Treg-associated cytokines, we also observed an increased mRNA expression of Th17 associated cytokines including the effector cytokine IL17 in DSS treated mice with *B. breve* intervention as compared to DSS treatment alone. It should be noted that Th17 cells are not the only source of IL17 production as it was demonstrated that also innate lymphoid cells can produce IL17 upon activation by IL23 derived from macrophages and dendritic cells [45] [46]. RORγt is the master transcription factor of Th17 cells [5], but is also expressed in IL17-producing innate lymphoid cells (ILC) [47]. We did not observe an effect of either DSS or treatment with *B.* breve on the expression of *Rorc* in the colon. The increased expression of *Il17* in the colon observed after *B. breve* intervention might be the result of IL23 mediated activation of resident ILC that are mainly found in the lamina propria in close proximity of epithelial cells. However, another possibility is that another, RORγt independent, IL17 producing source is present in the colon, such as B cells [48]. Although IL17 is often thought to promote the development of IBD [49], a recent study has demonstrated a protective function of IL17 in intestinal inflammation [50]. The exact role of IL17 during the IBD development still needs to be elucidated in additional studies.

Treg cells are associated with anti-inflammatory and tolerance inducing mechanisms [12], [51]. Although it is not totally clear how Treg cells effect the development of IBD, lack of Treg cells are often found in IBD patients [14], [15]. Animal models of IBD have further demonstrated the importance of Treg cells during the development of colitis [17], [33]. Foxp3 expression is associated with Treg cell development [6], [7] and the anti-inflammatory properties of Foxp3+ Treg cells have been demonstrated by a number of studies in both mice and humans [10], [52]. In this study, *B. breve* stimulation induced Treg cell differentiation *in vitro* and *in vivo.* Moreover, *B. breve* intervention ameliorated DSS-induced colitis symptoms and increased Foxp3+ T cells in Peyer's patches. Recent studies have demonstrated that the home of Peyer's patches, the small intestine, is involved in DSS colitis [53], [54]. Peyer's patches, like other lymphoid organs, contain dendritic cells that taken up antigens and present them to T cells, leading to T cell activation and differentiation. The increased Foxp3+ T cells in Peyer's patches indicate T cell differentiation that favors anti-inflammatory response. The resulting activated and expanded T cells have the potential to travel to the colon and induce immune regulation [55], [56], [57]. In line with this hypothesis, an increased amount of Foxp3+ cells was found in the colon of mice with *B. breve* intervention. These data suggest that the protective effects of *B. breve* on DSS colitis might lie with its capability to induce Treg cells

In conclusion, *B. breve* NutRes 204 stimulation *in vitro* leads to T cell skewing toward Treg cells in human PBMCs. Additionally; intervention with *B. breve* NutRes 204 ameliorated DSS-induced colitis symptoms *in vivo* with an increased amount of Treg cells and a reduced amount of Th17 cells in the GALT. This suggests that patients suffering from IBD could potentially benefit from *B. breve* intervention.

## Supporting Information

Figure S1 **FACS dot plots of T cell composition in human PBMCs with or without bacteria intervention.** FACS dot plots of A) fluorescence minus one (FMO) controls and B) isotype controls of FOXP3, RORγ, GATA3 and Tbet staining antibodies within CD4+ T cells are shown. Representative FACS dot plots of Th2 (GATA3+Tbet-) and Th1 (GATA-Tbet+), Th17 (RORγ+FOXP3-) and Treg (RORγ-FOXP3+) cells in the PBMCs after 48 hours incubation with either C) anti-CD3 stimulation alone, D) a combination of anti-CD3 and *L. rhamnosus*, or E) a combination of anti-CD3 and *B. breve* are illustrated. The percentage of activated CD4+CD69+ T cells is calculated within total live cells and the percentage of Th2, Th1, Th17 and Treg cells are shown within activated CD4+CD69+ T cell population.
(PDF)

Figure S2 **FACS dot plots of T cell – associated cytokine producing T cells in human PBMCs with or without bacteria intervention.** A) FACS dot plots of A) fluorescence minus one (FMO) controls and B) isotype controls of IL4, IL17, IL10 and IFNγ staining antibodies within CD4+ T cells are shown. Representative FACS dot plot of CD4+ T cells in the PBMCs after 7 days stimulation with anti-CD3 alone, or a combination of anti-CD3 with either *L. rhamnosus* or *B. breve* are shown in C). Gated on the CD4+ T cells, the percentages of D) IL4+, E) IL17+, F) IL10+ and G) IFNγ+ CD4+ T cells were determined. The percentage of CD4+ T cells is calculated within total live cells and the percentages of IL4+, IL17+, IL10+ and IFNγ+ CD4+ T cells are presented within CD4+ T cell population.
(PDF)

Figure S3 **FACS dot plots of Treg cells and Th17 cells in the mice with or without** ***B. breve*** **intervention.** A) FACS dot plots of A) fluorescence minus one (FMO) controls and B) isotype controls of Foxp3 and RORγt staining antibodies within CD4+ T cells are shown. Representative FACS dot plots of CD4+ cells in the Peyer's patches obtained from both healthy and DSS-treated mice, with or without *B. breve* intervention, are shown in C). Gated on CD4+ T cells, the percentages of D) Th17 (CD4+RORγt+Foxp3-) and Treg (CD4+RORγt-Foxp3+) cells were determined. The percentage of CD4+ T cells is calculated within total live cells and the percentages of Th17 and Treg cells are determined within CD4+ T cell population.
(PDF)
