# Supplementary figures and images for: Bifidobacterium breve Attenuates Murine Dextran Sodium Sulfate-Induced Colitis and Increases Regulatory T Cell Responses
Source: PLoS One. 2014 May 2;9(5):e95441. doi: 10.1371/journal.pone.0095441 (PMC4008378; doi:10.1371/journal.pone.0095441)

Figure S1

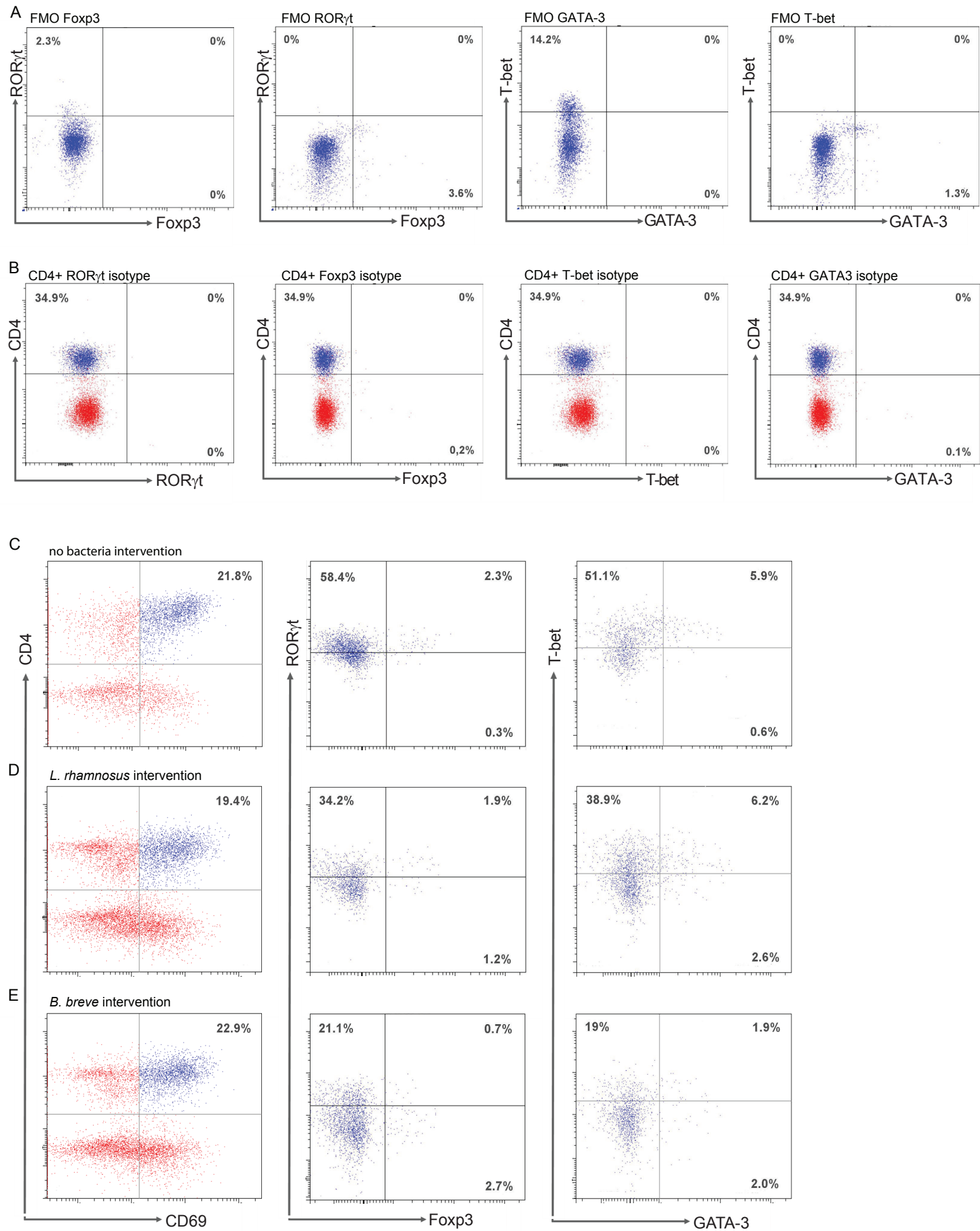

Supplement: Figure S1 — FACS dot plots of T cell composition in human PBMCs with or without bacteria intervention. FACS dot plots of A) fluorescence minus one (FMO) controls and B) isotype controls of FOXP3, RORγ, GATA3 and Tbet staining antibodies within CD4+ T cells are shown. Representative FACS dot plots of Th2 (GATA3+Tbet-) and Th1 (GATA-Tbet+), Th17 (RORγ+FOXP3-) and Treg (RORγ-FOXP3+) cells in the PBMCs after 48 hours incubation with either C) anti-CD3 stimulation alone, D) a combination of anti-CD3 and L. rhamnosus, or E) a combination of anti-CD3 and B. breve are illustrated. The percentage of activated CD4+CD69+ T cells is calculated within total live cells and the percentage of Th2, Th1, Th17 and Treg cells are shown within activated CD4+CD69+ T cell population. (PDF) [file pone.0095441.s001.pdf]

Figure S2

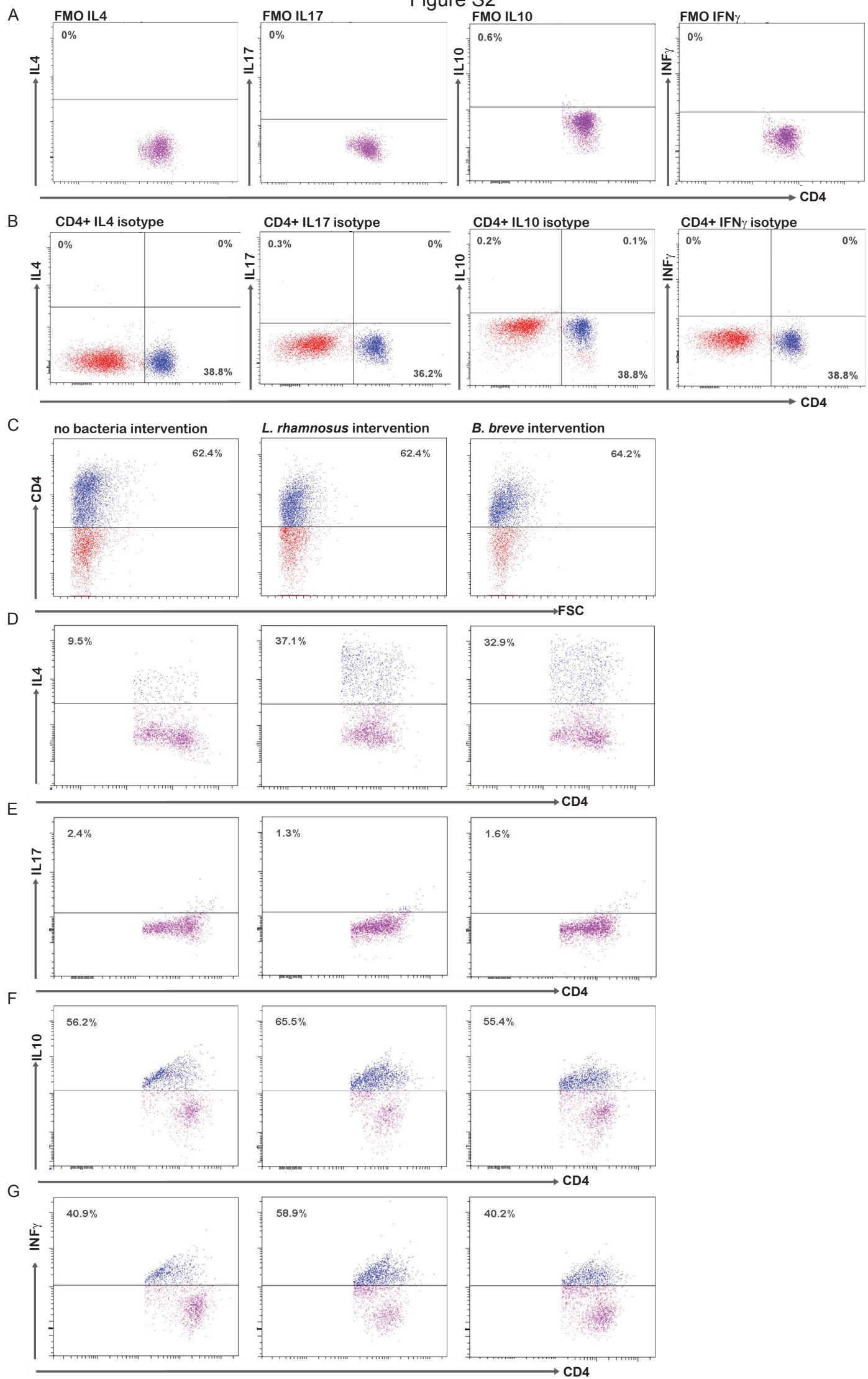

Supplement: Figure S2 — FACS dot plots of T cell – associated cytokine producing T cells in human PBMCs with or without bacteria intervention. A) FACS dot plots of A) fluorescence minus one (FMO) controls and B) isotype controls of IL4, IL17, IL10 and IFNγ staining antibodies within CD4+ T cells are shown. Representative FACS dot plot of CD4+ T cells in the PBMCs after 7 days stimulation with anti-CD3 alone, or a combination of anti-CD3 with either L. rhamnosus or B. breve are shown in C). Gated on the CD4+ T cells, the percentages of D) IL4+, E) IL17+, F) IL10+ and G) IFNγ+ CD4+ T cells were determined. The percentage of CD4+ T cells is calculated within total live cells and the percentages of IL4+, IL17+, IL10+ and IFNγ+ CD4+ T cells are presented within CD4+ T cell population. (PDF) [file pone.0095441.s002.pdf]

**Figure S3**

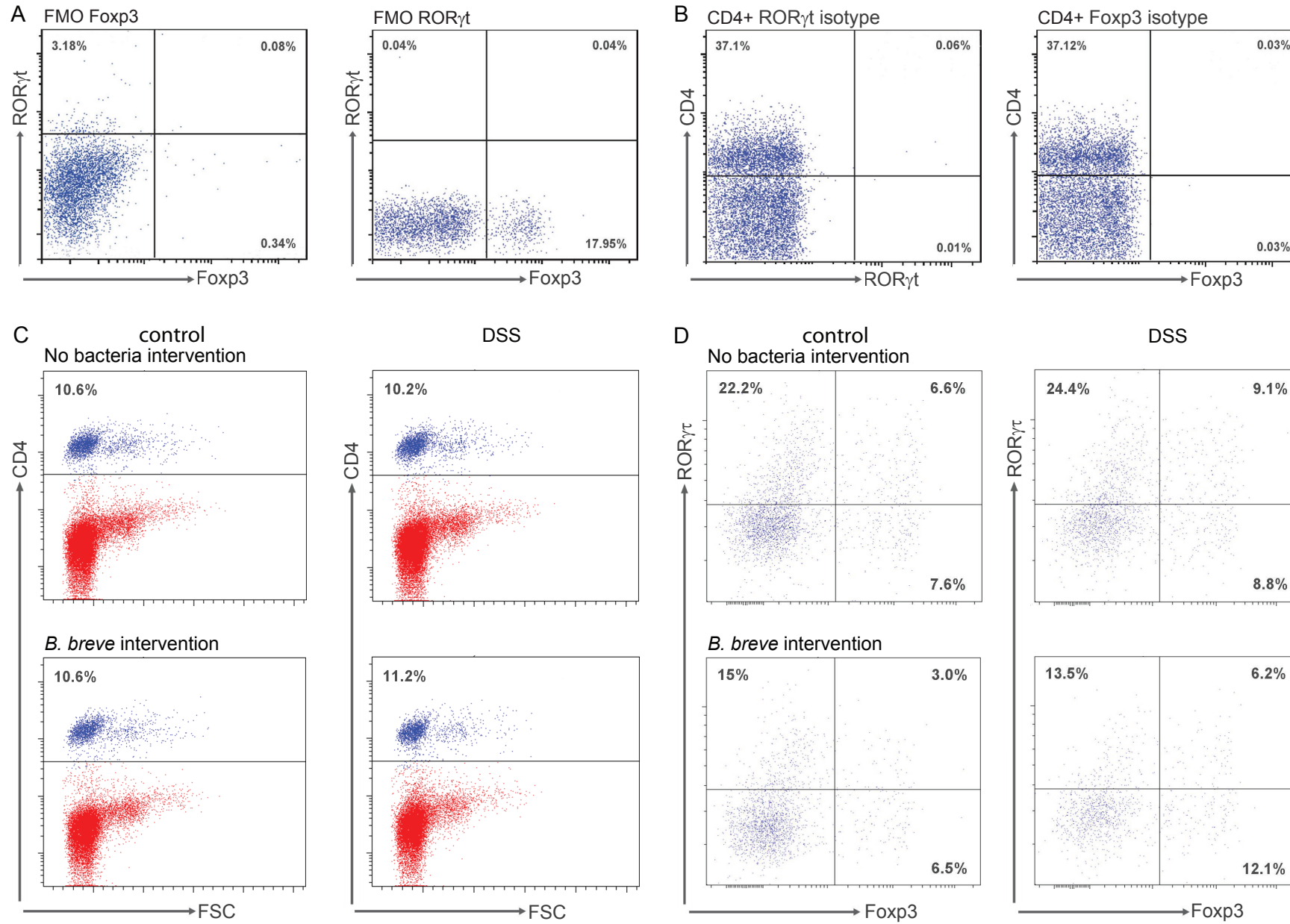

Supplement: Figure S3 — FACS dot plots of Treg cells and Th17 cells in the mice with or without B. breve intervention. A) FACS dot plots of A) fluorescence minus one (FMO) controls and B) isotype controls of Foxp3 and RORγt staining antibodies within CD4+ T cells are shown. Representative FACS dot plots of CD4+ cells in the Peyer's patches obtained from both healthy and DSS-treated mice, with or without B. breve intervention, are shown in C). Gated on CD4+ T cells, the percentages of D) Th17 (CD4+RORγt+Foxp3-) and Treg (CD4+RORγt-Foxp3+) cells were determined. The percentage of CD4+ T cells is calculated within total live cells and the percentages of Th17 and Treg cells are determined within CD4+ T cell population. (PDF) [file pone.0095441.s003.pdf]
